# Relationship of CT-quantified emphysema, small airways disease and bronchial wall dimensions with physiological, inflammatory and infective measures in COPD

**DOI:** 10.1186/s12931-018-0734-y

**Published:** 2018-02-20

**Authors:** Kristoffer Ostridge, Nicholas P. Williams, Viktoriya Kim, Stephen Harden, Simon Bourne, Stuart C. Clarke, Emmanuel Aris, Sonia Mesia-Vela, Jeanne-Marie Devaster, Andrew Tuck, Anthony Williams, Stephen Wootton, Karl J. Staples, Tom M. A. Wilkinson, on behalf of the AERIS Study Group

**Affiliations:** 1 Clinical and Experimental Sciences, Faculty of Medicine, University of Southampton, Southampton, UK; 2 NIHR Southampton Biomedical Research Centre, University Hospital Southampton NHS Foundation Trust, Southampton, UK; 3 Department of Radiology, University Hospital Southampton NHS Foundation Trust, Southampton, UK; 4 Portsmouth Hospitals NHS Trust, Queen Alexandra Hospital, Portsmouth, UK; 5 Wessex Investigational Sciences Hub, University of Southampton Faculty of Medicine, Southampton General Hospital, Southampton, UK; 6 GSK Vaccines, Rixensart, Belgium; 7 Faculty of Medicine and Institute for Life Sciences, University of Southampton, Southampton, UK

**Keywords:** COPD, CT, Imaging, Phenotyping, Emphysema

## Abstract

**Background:**

COPD is a complex, heterogeneous disease characterised by progressive development of airflow limitation. Spirometry provides little information about key aspects of pathology and is poorly related to clinical outcome, so other tools are required to investigate the disease. We sought to explore the relationships between quantitative CT analysis with functional, inflammatory and infective assessments of disease to identify the utility of imaging to stratify disease to better predict outcomes and disease response.

**Methods:**

Patients from the AERIS study with moderate-very severe COPD underwent HRCT, with image analysis determining the quantity of emphysema (%LAA_<− 950_), small airways disease (E/I MLD) and bronchial wall thickening (Pi10). At enrolment subjects underwent lung function testing, six-minute walk testing (6MWT), blood sampling for inflammatory markers and sputum sampling for white cell differential and microbiological culture and PCR.

**Results:**

122 subjects were included in this analysis. Emphysema and small airways disease had independent associations with airflow obstruction (β = **−** 0.34, *p* < 0.001 and β = − 0.56, p < 0.001). %LAA_<− 950_ had independent associations with gas transfer (β = **−** 0.37, p < 0.001) and E/I MLD with RV/TLC (β = 0.30, p =0.003). The distance walked during the 6MWT was not associated with CT parameters, but exertional desaturation was independently associated with emphysema (β = 0.73, p < 0.001). Pi10 did not show any independent associations with lung function or functional parameters.

No CT parameters had any associations with sputum inflammatory cells. Greater emphysema was associated with lower levels of systemic inflammation (CRP β = − 0.34, p < 0.001 and fibrinogen β = − 0.28, p =0.003). There was no significant difference in any of the CT parameters between subjects where potentially pathogenic bacteria were detected in sputum and those where it was not.

**Conclusions:**

This study provides further validation for the use of quantitative CT measures of emphysema and small airways disease in COPD as they showed strong associations with pulmonary physiology and functional status. In contrast to this quantitative CT measures showed few convincing associations with biological measures of disease, suggesting it is not an effective tool at measuring disease activity.

**Electronic supplementary material:**

The online version of this article (10.1186/s12931-018-0734-y) contains supplementary material, which is available to authorized users.

## Background

Chronic obstructive pulmonary disease (COPD) is a heterogeneous disease characterised by the progressive development of airflow limitation, leading to functional impairment and associated symptoms [1]. The clinical features and natural history of the condition vary considerably and traditional methods of measuring airflow obstruction do not reflect the complexity of the condition or the underlying pulmonary pathology. This has limited our understanding of COPD and consequently, there have not been the improvements in outcome seen in other chronic diseases and there are no significant disease-modifying medications. Other tools are required to help explain the heterogeneity and provide further insights into approaches to study and manage the condition.

Computed tomography (CT) can image the key pathological changes seen in COPD, including emphysema and remodelling of the large and small airways [2]. These pathologies both contribute to airflow obstruction and therefore CT has the potential to provide vital insights into the exact nature of the underlying pathophysiology. Newly developed automated image assessment techniques enable segmentation of the lung parenchyma and airways from the chest wall and surrounding structures, thus allowing quantitative analysis of emphysema, bronchial wall dimensions and small airways disease to characterise this heterogeneous disease more effectively.

Emphysema can be visualised as low attenuation areas on CT and by applying a density mask to the lung parenchyma, the percentage of voxels below − 950 Hounsfield (%LAA_<− 950_) can be used to assess the quantity of emphysema. This method shows strong associations with histological measures of emphysema as well as physiological markers of disease [3–5]. Using CT imaging, three-dimensional reconstructions of the bronchial tree can be generated down to the fifth or sixth generation airway, allowing analysis of airway wall dimensions. Thus far, studies have shown equivocal results, with some demonstrating thicker airway walls in COPD [6–8] and others demonstrating thinner walls [3, 9]. This may be partly explained by the variation in methods used to describe airway wall dimensions and therefore a standardised parameter called Pi10 has been developed. This predicts the square root of the wall area for a hypothetical airway with an internal perimeter of 10 mm, giving a single value for bronchial wall thickening of the entire bronchial tree and has shown an inverse correlation with forced expiratory volume in one second (FEV1) [3]. Due to limited resolution, CT cannot image the small airways directly and instead quantification of air trapping can be used as a surrogate marker. A number of CT-derived methods exist to measure air trapping with one of the most widely used being the ratio of the mean lung density (MLD) in expiration to inspiration. In COPD, this measure has been found to correlate with a number of physiological and functional parameters [10–12].

Recent analysis from large COPD cohort studies have added significantly to our understanding of the use of quantitative CT imaging in COPD, in particular exploring the relationships with pulmonary physiology [3, 13], functional ability [14, 15] and tracking the longitudinal progression of disease over time [13]. However, these large-scale studies allow only limited characterisation of their cohorts, with fewer detailed assessments possible. Furthermore, few studies have assessed biological markers of COPD, including measures of inflammation and infection. This further work is required to confirm CT analysis as an imaging biomarker of disease that can be a useful research tool to provide insights into the disease and as a clinical measure to guide management of patients with COPD. In the present study we performed quantitative CT analysis on a well-characterised cohort of patients with moderate to very severe COPD and explored the relationship with multiple clinical, physiological, microbiological and inflammatory indices to identify the utility of imaging to stratify disease to better predict outcomes and disease response.

## Methods

### Study design and study population

The Acute Exacerbation and Respiratory Infections in COPD (AERIS) study (ClinicalTrials.gov: NCT01360398, GSK study number 114378) is a prospective, observational cohort study and has been described in detail elsewhere [16, 17]. Briefly, subjects with moderate to very severe COPD, as defined by GOLD guidelines [1] were recruited. Subjects had at least a 10 pack year smoking history and had suffered at least one moderate or severe exacerbation in the preceding 12 months. Exclusion criteria included a history of other pulmonary disease, long-term antibiotics/systemic steroids or an exacerbation within the month prior to recruitment. All Subjects gave written informed consent and the study was approved by the South Central - Southampton B NRES Committee. We report a secondary analysis focusing on CT results at enrolment.

### Procedures

At enrolment subjects had full clinical assessment, CT imaging, pulmonary function testing, six-minute walk testing, blood and sputum sampling.

### CT scanning and quantitative image analysis

Subjects underwent volumetric CT scans of the chest using a Siemens Sensation 64 CT scanner. The imaging protocol consisted of; slice thickness 0.75 mm, slice separation 0.5 mm, tube voltage 120KV, effective mAs 90mAs (using dose modulation), collimation 0.6 mm and a pitch of 1. Subjects were scanned at full inspiration and at maximum expiration. These images were assessed by an experienced thoracic radiologist who visually reported the presence of bronchiectasis. This was defined as either a non-tapering bronchus with an internal diameter over 110% than the adjacent pulmonary artery or the presence of a visible bronchi within 1 cm of the pleural surface. The bronchiectasis score was determined in each lobe using a previously validated scoring system [18]; 0 if no bronchiectasis was present, 1 if less than 25% of bronchi were bronchiectatic, 2 for 25–49%, 3 for 50–74% and 4 for over 75%. An overall score was calculated out of maximum of 24 (the lingual was included as a separate lobe). Images reconstructed with the B35 kernel were used for image analysis using Apollo Software (VIDA Diagnostics). Emphysema was quantified by the percent of lung voxels on the inspiratory scan with attenuation values below -950HU (%LAA_<− 950_). Bronchial wall thickening was quantified using the standardised parameter Pi10, which is the square root of the wall area of a hypothetical airway with a 10 mm internal perimeter. A surrogate marker for small airways disease was measured using the ratio of mean lung attenuation on expiratory and inspiratory scans (E/I MLD).

### Pulmonary function and six-minute walk tests

Pre and post-bronchodilator spirometry, plethysmography and gas transfer were performed in accordance with ATS guidelines [19]. The 6MWT was performed on a standard 30-m course and the patient was instructed to walk round the course at their own pace for 6 min and the number of laps walked in 6 min recorded. Finger-tip pulse oximetry was used to measure baseline and lowest saturations.

### Sputum and blood testing

Sputum samples were obtained by spontaneous expectoration or by induction and were processed according to standard methods, described previously [20]. Briefly, sputum was collected in a petri dish and processed within 2 h. Sputum plugs were separated from saliva using tweezers and samples were divided for biomarker and microbiology analysis. Samples were sent to the Public Health England laboratory for culture-based microbiology. Potential bacterial respiratory pathogens, including *Haemophilus influenzae (NTHI)*, *Moraxella catarrhalis (MCAT)*, *Streptococcus pneumoniae (SP)*, *Pseudomonas aeruginosa (PA)*, and *Staphylococcus aureus (SA)* were identified using conventional culture techniques and PCR, described previosuly [17]. Sputum for white cell differential analysis was filtered through 100 μm filters and centrifuged at 400 g for 10 min at 4 °C. The resulting cell pellet was resuspended in PBS at a concentration of 5 x 105 cells/ml and were loaded onto poly-L-lysine coated glass slides using a cytocentrifuge. These were stained with Rapid Romanowsky stain (Raymond Lamb Ltd., Eastbourne, UK) and differential cell counts were obtained by counting 400 cells using light microscopy. For quality control purposes, only sputum samples with fewer than 30% squamous cells were included in the analysis.

Phlebotomy was performed and samples were processed by conventional methods for full blood count, C-reactive protein (CRP), fibrinogen and pro-calcitonin (PCT).

### Statistical analysis

Statistical analyses were performed using SPSS version 23. The differences in demographic, physiological, biological and CT parameters between GOLD groups (2010, spirometric criteria) were tested using the Kruskal-Wallis test. Univariate associations between these parameters were assessed using Spearman’s correlation with rho and *p* values presented. FEV1%, TLCO%, RV/TLC, 6MWD, desaturation on exertion, CRP, fibrinogen, and sputum neutrophils/eosinophils were analysed via multiple linear regression on dependent variables such as CT parameters %LAA_<− 950_, E/I MLD and demographic variables (age, gender, current smoking status, pack years and BMI). Only variables that made a significant difference to the model are included in the results, with variables chosen using forward selection. Differences in CT parameters and FEV1% between subjects who could walk more or less than 350 m or did/did not desaturate at the 6MWT were tested using the Mann Whitney U test and logistic regression was used to conduct multivariate analysis. Mann Whitney U test was also used to assess the differences in CT parameters between subjects who did or did not culture bacteria in their sputum. Throughout the analysis a *p*-value of < 0.05 was considered statistically significant. Since the comparisons presented here are not part of the primary and secondary objectives of the AERIS study, the results should be considered as post hoc.

## Results

Of 152 patients screened, 127 were enrolled onto the study. 122 of these had complete CT data as 5 of the scans could not be quantitatively analysed fully for technical reasons. All 122 had complete data for demographics, spirometry and CRP. 117 subjects had gas transfer data, 113 had plethysmography data, 120 had 6MWT parameters, 121 blood white cells and procalcitonin and 109 had fibrinogen. 106 subjects had sufficient sputum collected for microbiology culture while 98 had microbiology PCR performed. As sputum was prioritised for microbial analysis, only 75 subjects had enough for white cell differential and of these, 66 were deemed good quality samples (< 30% squamous cells). Baseline data for demographics, pulmonary physiology, biological and CT data are shown in Table 1. The majority of patients had moderate or severe airflow obstruction. There was no significant difference in age, gender, BMI or smoking status between the GOLD groups. As expected there was significantly lower FEV1% and FEF75–25% in the more severe GOLD groups. There was also significantly lower gas transfer and higher RV and RV/TLC ratio in subjects with severe and very severe COPD compared to moderate COPD. Subjects with very severe COPD had significantly lower 6MWD than subjects with either moderate or severe COPD. Subjects with severe and very severe COPD desaturated during exertion significantly more than those with moderate disease. There was no significant difference in sputum or blood inflammatory markers between the GOLD groups.

**Table 1:** Characteristics of study subjects included in the study

	*N* = 122	GOLD 2 (*N* = 56)	GOLD 3 (*N* = 49)	GOLD 4 (*N* = 17)	*P* value
Age (years)	67.0 (11.0)	67.5 (13)	67.0 (10)	65.0 (10)	0.992
Male	67	31	25	11	0.618
BMI (kg/m^2^)	27.1 (6.9)	27.7 (7.3)	26.5 (8.0)	25.4 (7.2)	0.054
Current smoker	46	21	18	7	0.947
Pulmonary Function					
FEV1%	47.2 (23.7)	59.1 (12.9) #^	38.8 (10.5)*^	26.2 (5.9)*#	< 0.001
FEV1/FVC	0.43 (0.12)	0.50 (0.10) #^	0.38 (0.15) *^	0.29 (0.08) *#	< 0.001
FEF75–25%	13.2 (8.3)	17.2 (6.4) #^	11.0 (4.7) *^	6.8 (2.2) *#	< 0.001
TLCO%	58.3 (28.7)	70.4 (23.9) #^	53.0 (22.8)*	44.6 (11.8)*	< 0.001
RV%	144.8 (59.2)	133.8 (38.9) #^	159.2 (48.1)*	202.7 (96.6)*	< 0.001
RV/TLC	0.54 (0.14)	0.49 (0.14) #^	0.58 (0.10)*	0.64 (0.16)*	< 0.001
Functional Markers					
6MWD (meters)	303.5 (174)	360.0 (155)^	300.0 (156)^	169.5 (129)*#	< 0.001
Desaturation on Exertion (%)	4.0 (5.0)	3.0 (3.0) #^	5.0 (8.0)*	6.0 (6.5)*	0.001
Blood Measures					
Blood neutrophils (10^9^L)	4.8 (1.6)	4.8 (1.6)	4.7 (2.0)	4.8 (1.6)	0.914
Blood eosinophils (10^9^L)	0.20 (0.20)	0.20 (0.20)	0.20 (0.20)	0.20 (0.20)	0.082
Fibrinogen (g/L)	4.8 (1.1)	4.8 (1.1)	4.8 (1.0)	5.0 (1.4)	0.813
CRP (mg/L)	5.0 (7.0)	5.0 (6.8)	4.0 (6.5)	6.0 (7.0)	0.570
Procalcitonin (μg/L)	0.059 (0.03)	0.056 (0.02)	0.061 (0.03)	0.073 (0.05)	0.236
Sputum Differential					
Sputum Neutrophils(%)	45.9 (70.8)	17.7 (62.6)	44.3 (75.8)	71.2 (39.3)	0.110
Sputum Eosinophils (%)	2.02 (5.2)	1.97 (4.0)	2.20 (5.7)	2.62 (4.8)	0.596
CT Measures					
Emphysema (%LAA_<− 950_)	12.0 (20.7)	9.6 (13.1)#^	15.8 (26.9)*	24.8 (12.3)*	< 0.001
Bronchial wall area (Pi10)	3.80 (0.12)	3.79 (0.13)	3.78 (0.10)^	3.84 (0.05)#	0.040
Small airways disease (E/I MLD)	0.92 (0.07)	0.88 (0.07) #^	0.93 (0.05)*	0.96 (0.03)*	< 0.001
Bronchiectasis	8 (6.6)	1 (1.8)	5 (10.2)	2 (11.8)	0.143

Values given as medians (IQR). Males, smoking status and bronchiectasis given as number of subjects. *N* = 122 for whole cohort. For gas transfer *n* = 117, plethysmography *n* = 113, 6MWT parameters *n* = 120, blood white cells and procalcitonin *n* = 121, fibrinogen *n* = 109, sputum white cell differential *n* = 66. *P* value indicates difference between GOLD groups where < 0.05 taken as significant. * significant difference vs. GOLD 2 group, # significant difference vs. GOLD 3 group and ^ significant difference vs. GOLD 4 group

When assessing the CT parameters, there was significantly more emphysema and air trapping in severe and very severe COPD subjects compared to subjects with moderate COPD (Table 1). Pi10 was significantly raised in very severe COPD compared to subjects with severe COPD. Only 8 subjects had clinically significant bronchiectasis present on their CT scans and even then this was relatively mild with a median bronchiectasis score of 2.5. Given this low number of subjects with bronchiectasis, further analysis was not possible on this CT parameter. There was a significant positive association between %LAA_<− 950_ and E/I MLD (rho = 0.47, *p* < 0.001) (Additional file 1: Figure S1). There was also an inverse relationship between %LAA_<− 950_ and Pi10 (rho = − 0.36, *p* < 0.001) and a much weaker association between E/I MLD and Pi10 (rho = 0.18, p 0.045).

### Relationship between CT parameters, PFTs and functional markers of disease

**Fig. 1:**
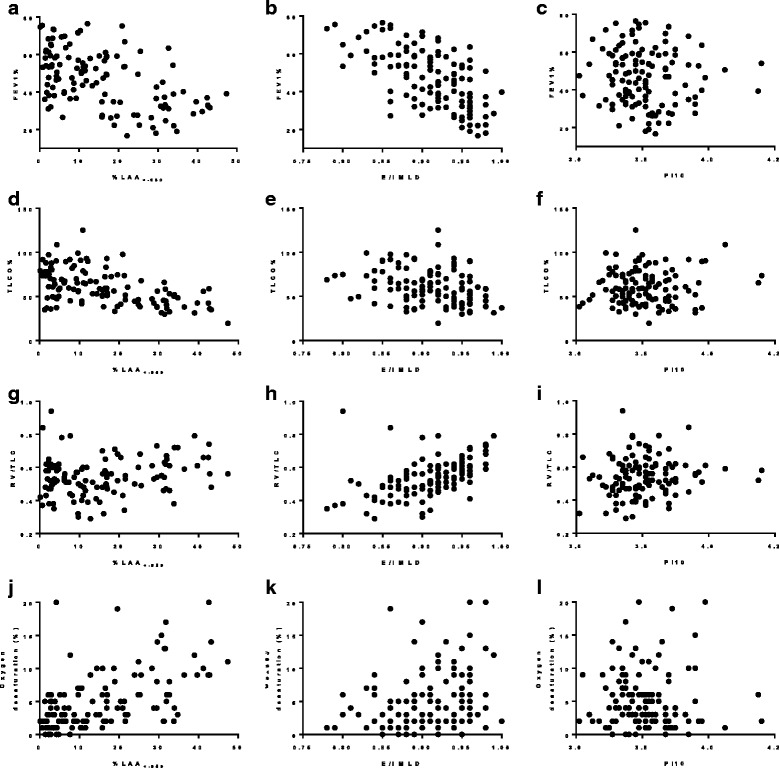
Scatterplots of (**a**) %LAA_<−950_ against FEV1% (rho −0.49***) (**b**) E/I MLD against FEV1% (rho −0.63***) (**c**) Pi10 against FEV1% (rho −0.11) (**d**) %LAA_<− 950_ against TLCO% (rho − 0.51***) (**e**) E/I MLD against TLCO% (rho − 0.34***) (**f**) Pi10 against TLCO% (rho 0.01) (**g**) %LAA_<− 950_ against RV/TLC (rho 0.19*) (**h**) E/I MLD against RV/TLC (rho 0.62***) (**i**) Pi10 against RV/TLC (rho 0.16) (**j**) %LAA_<− 950_ against oxygen desaturation (rho 0.56***) (**k**) E/I MLD against oxygen desaturation (rho 0.27**) (**l**) Pi10 against oxygen desaturation (rho − 0.11). For comparisons with FEV1% N = 122, for TLCO% N = 117, for RV/TLC N = 113 and for oxygen desaturation *N* = 120. * *p* < 0.05, ** *p* < 0.01, *** *p* < 0.001

There were strong negative associations between airflow obstruction and %LAA_<− 950_ and E/I MLD (Table 2 and Fig. 1). In contrast to this there was no significant association between FEV1% and Pi10. On multivariate analysis only %LAA_<− 950_, E/I MLD and BMI had independent associations with FEV1% (Table 3). %LAA_<− 950_ and E/I MLD also had significant associations with gas transfer. %LAA_<− 950_ was the only CT parameter which had significant independent associations with gas transfer on multivariate analysis. %LAA_<− 950_ and E/I MLD also had significant associations with RV% and RV/TLC ratio. On multivariate analysis only E/I MLD, FEV1% and gender significantly predicted RV/TLC ratio.

**Table 2:** Spearman’s correlation analysis between CT parameters, pulmonary function tests, functional markers, blood and sputum markers

	%LAA_<− 950_	E/I MLD	Pi10
Pulmonary Function Tests			
FEV1%	−0.49***	−0.63***	− 0.11
FVC%	0.06	−0.21*	− 0.31**
FEV1/FVC	−0.72***	− 0.64***	0.12
TLCO%	−0.51***	−0.34***	0.01
RV%	0.36***	0.64***	−0.05
RV/TLC	0.19*	0.62***	0.16
Functional Markers			
6MWD (meters)	−0.14	−0.10	− 0.11
Desaturation on exertion (%)	0.56***	0.27**	−0.11
Blood Markers			
Blood neutrophils (10^9^L)	−0.06	−0.11	0.15
Blood eosinophils (10^9^L)	0.15	0.01	0.01
Fibrinogen (g/L)	−0.20*	− 0.04	0.21*
CRP (mg/L)	−0.27**	−0.28**	0.25*
Procalcitonin (μg/L)	−0.01	−0.06	0.18*
Sputum Differential			
%neutrophils	0.17	0.19	−0.10
%eosinophils	0.16	0.04	−0.11

Spearman’s r values given. N = 122 apart from associations with gas transfer (n = 117), plethysmography (n = 113), 6MWT parameters (n = 120), blood white cells and procalcitonin (n = 121), fibrinogen (n = 109) and sputum white cell differential (n = 66). *p < 0.05, **p < 0.01, ***p < 0.001

**Table 3:** Multiple regression analysis predicting FEV1%, TLCO%, RV/TLC, 6MWD, desaturation on exertion, CRP, fibrinogen and sputum neutrophils

	B coefficient	Standardised B coefficient	significance
FEV1%			
%LAA_<− 950_	− 0.42	− 0.34	< 0.001
E/I MLD	− 180.8	− 0.56	< 0.001
BMI	−0.56	− 0.20	0.010
TLCO%			
%LAA_<− 950_	−0.62	−0.37	< 0.001
FEV1%	0.49	−0.39	< 0.001
Gender	−7.5	−0.19	0.014
RV/TLC			
E/I MLD	0.75	0.30	0.003
FEV1%	−0.003	−0.32	0.002
Gender	0.07	0.28	< 0.001
6MWD (metres)			
FEV1%	3.03	0.41	< 0.001
BMI	−5.19	−0.26	0.003
Gender	−38.2	−0.17	0.044
Desaturation on exertion (%)			
%LAA_<−950_	0.041	0.73	< 0.001
BMI	0.032	0.248	0.004
Gender	0.233	0.164	0.037
CRP (mg/L)			
%LAA_<− 950_	−0.026	−0.34	< 0.001
Fibrinogen (g/L)			
%LAA_<−950_	−0.020	−0.28	0.003
Age	0.022	0.216	0.020
Sputum Neutrophils (%)			
Current smoker	−21.1	−0.29	0.018

%LAA_<−950_, E/I MLD, PI10, bronchiectasis, FEV1% and demographics variables (age, gender, current smoking status, pack years and BMI) were combined into a regression model to predict each of the dependent variables. Only variables that made a significant difference to the model are included in the results. The natural log of CRP and desaturation on exertion (+ 1) were used for this analysis to normalise the distribution of the residuals

6MWD did not have any significant associations with any CT parameters (Table 2). On multivariate analysis only FEV1%, gender and BMI showed an independent association with 6MWD (Table 3). There was also no difference in CT parameters between subjects who could walk more than 350 m compared to those that could not (Table 4). On logistic regression analysis higher FEV1 and being male were the only variables that predicted being able to walk over 350 m (Table 6). Desaturation on exertion was significantly associated with both %LAA_<− 950_ and E/I MLD (Table 2 and Fig. 1). On multivariate analysis %LAA_<− 950_, BMI and gender were independently associated with desaturation (Table 3). Subjects who desaturated > 5% had a significantly raised %LAA_<− 950_ and E/I MLD and significantly lower FEV1%, but showed no difference in Pi10 (Table 5). On logistic regression analysis only higher %LAA_<− 950_ predicted whether a subject desaturated more than 5% (Table 6).

**Table 4:** CT parameters and FEV1% in patient who could walk more or less than 350 m at the 6MWT

	< 350 m (*N* = 75)	> 350 m (*N* = 45)	*P* Value
FEV1%	40.1 (24.9)	53.4 (14.9)	0.002
%LAA_<− 950_	15.8 (24.1)	9.7 (13.9)	0.027
E/I MLD	0.92 (0.07)	0.91 (0.07)	0.150
Pi10	3.81 (0.12)	3.79 (0.12)	0.641

Values represent medians and IQR. *P* value tested using Mann Whitney U test

**Table 5:** CT parameters and FEV1% in patient who desaturated or not at 6MWT

	< 5% desaturation (*N* = 76)	> 5% desaturation (*N* = 44)	*P* Value
FEV1%	52.9 (19.2)	36.5 (18.4)	< 0.001
%LAA_<− 950_	7.9 (13.5)	24.9 (19.3)	< 0.001
E/I MLD	0.91 (0.07)	0.94 (0.06)	0.010
Pi10	3.91 (0.12)	3.78 (0.11)	0.232

Values represent medians and IQR. *P* value tested using Mann Whitney U test

**Table 6:** Logistic regression predicting which variables contributed to patients walking over 350 m or desaturating during the 6MWT

	Odds ratio	95% Lower CI	95% upper CI	significance
6MWD > 350 m				
FEV1%	1.049	1.019	1.079	0.001
Female	0.379	0.165	0.871	0.022
Desaturation > 5%				
%LAA_<−950_	1.101	1.059	1.143	< 0.001

44 subjects desaturated > 5% and 76 subjects did not

45 subjects walked over 350 m and 75 subjects did not

%LAA_<− 950_, E/I MLD and PI10, FEV1% and demographics variables (age, gender, current smoking status, pack years and BMI) were combined into a regression model to predict each of the dependent variables. Only variables that made a significant difference to the model are included in the results

### Relationship between CT parameters and blood and sputum inflammatory markers and sputum microbiology

There were no significant associations between CT parameters and blood neutrophils and eosinophils (Table 2). Emphysema had a significant inverse relationship with CRP and fibrinogen, while E/I MLD had an inverse association with CRP. In addition, Pi10 had a positive association with fibrinogen, CRP and procalcitonin. On multivariate analysis only %LAA_<− 950_ had a weak independent relationship with CRP and fibrinogen (Table 3).

There were no associations between sputum neutrophils and eosinophils and any of the quantitative CT measures (Table 2 and Table 3) To ensure that only using good quality sputum samples (squamous cells < 30%) did not bias the results the analysis was repeated with all samples and similar results were found (Additional file 1: Table S1).

There were also few differences in emphysema, air trapping and Pi10 values between subjects who cultured bacteria in their sputum and those that did not (Table 7). The only exceptions to this was that Pi10 was significantly higher in those that did not have SA in their sputum compared to these that did and E/I MLD was significantly higher in those that cultured PA compared to those that didn’t. When repeating the analysis using PCR to detect the presence of bacteria in sputum the results were much the same, with only E/I MLD being significantly higher in those that had PA detected compared to those that did not (Additional file 1: Table S2).

**Table 7:** CT parameters in subjects according to sputum microbiology

	Frequency	%LAA_<−950_	E/I MLD	Pi10
Potentially Pathogenic Bacteria				
Culture positive	55	10.9 (17.2)	0.92 (0.07)	3.79 (0.12)
Culture negative	51	15.1 (24.0)	0.90 (0.07)	3.78 (0.09)
P value	–	0.260	0.152	0.146
Haemophilus influenzae				
Culture positive	31	11.5 (18.0)	0.92 (0.08)	3.79 (0.11)
Culture negative	75	13.3 (20.0)	0.91 (0.07)	3.79 (0.10)
P value	–	0.370	0.207	0.197
Moraxella catarrhalis				
Culture positive	8	12.4 (23.1)	0.93 (0.07)	3.78 (0.10)
Culture negative	98	12.5 (20.3)	0.91 (0.07)	3.79 (0.11)
P value	–	0.981	0.720	0.549
Streptococcus pneumoniae				
Culture positive	18	10.2 (9.9)	0.92 (0.05)	3.83 (0.11)
Culture negative	88	14.3 (24.9)	0.91 (0.08)	3.79 (0.09)
P value	–	0.501	0.626	0.203
*Staphylococcus aureus*				
Culture positive	6	13.5 (22.4)	0.91 (0.08)	3.74 (0.05)
Culture negative	100	12.5 (20.6)	0.91 (0.07)	3.80 (0.11)
P value	–	0.667	0.682	0.039*
Pseudomonas aeruginosa				
Culture positive	6	21.4 (27.5)	0.95 (0.06)	3.83 (0.16)
Culture negative	100	12.0 (20.1)	0.91 (0.07)	3.79 (0.11)
P value	–	0.367	0.034*	0.498

Frequency given as number of subjects. For CT parameters, values represent medians and IQR. *P < 0.05 using Mann Whitney U test

## Discussion

In the AERIS study we used sophisticated, cutting-edge techniques to explore the heterogeneity of COPD, resulting in an extremely well-characterised cohort of subjects. This was complemented by using CT analysis to understand the key structural changes identified in COPD and describing the relationship with a number of key physiological, functional and biological markers of disease. The main findings from the present analysis were the demonstration of a novel independent association between emphysema and desaturation on exertion, but interestingly not on exercise capacity. We also showed that CT-derived measurements of emphysema and small airways disease were independently associated with FEV1%. These CT measures also had strong associations with other pulmonary function tests, with emphysema having the strongest association with gas transfer and small airways disease having the strongest association with RV/TLC. None of the CT parameters were associated with sputum inflammatory cells or microbiology.

In this study, subjects exhibited a wide range of emphysema and small airways disease, with those in more severe GOLD categories having larger amounts, which is consistent with previous studies [3]. For dimensions of larger airways this was less clear-cut with only those in the most severe GOLD group having larger airway wall dimensions than severe COPD but not moderate COPD. Other studies have also shown inconsistent results when using Pi10 as a marker of bronchial wall thickening in COPD [3, 9, 21]. Bronchiectasis was not particularly common in this cohort, which is inconsistent with previous work that have shown higher rates in COPD populations [18, 22, 23]. The reasons for this are unknown, however subjects from this study were recruited from an area with low TB prevalence and most patients were recruited from specialist COPD clinics where clinically relevant bronchiectasis would have been detected and referred to specialist care. When assessing the association between these CT measures, a significant positive association was found between emphysema and small airways disease. However, the scatterplots demonstrated significant variability of small airways disease at lower levels of emphysema. It has been hypothesised that small airways disease may be a precursor to emphysema although more longitudinal work is required to understand this. There was also a weak inverse association between Pi10 and emphysema. Interestingly there was no real relationship between Pi10 and small airways disease, suggesting that structural abnormalities of the large and small airways are unrelated in COPD.

CT measures of emphysema and small airways disease had independent associations with airflow obstruction, which is in agreement with previous studies [3]. E/I MLD had the strongest association with FEV1%, suggesting small airways disease is the largest contributor to airflow obstruction in COPD. Emphysema and small airways disease also had significant associations with gas transfer, although in multivariate analysis %LAA_<− 950_ was the only CT parameter had independent associations. As gas transfer is a measure of the disruption of the alveolar-capillary membrane it is unsurprising that emphysema was the only CT measure that showed this association and is consistent with prior studies [11, 24–30]. E/I MLD was the only CT measure to independently predict RV% and RV/TLC ratio. RV and RV/TLC ratio are measures of pulmonary air trapping, therefore confirming E/I MLD as a an accurate marker of small airways disease, which has also been shown by other studies [10, 24, 31, 32]. Pi10 did not show an independent associations with any lung function parameters.

It is vital to understand the pathologies that contribute to poor exercise in COPD, as lower exercise capacity during the 6MWT has been linked with increased mortality [33–37]. We found no associations between the 6MWD and CT parameters, with only FEV1%, BMI and gender showing an independent association. In addition, FEV1% was the only variable that significantly predicted which subjects walked less than 350 m. Previous work has suggested that subjects with increased emphysema [15, 27, 38, 39] and air trapping [11, 14, 38] walk shorter distances at the six minute walk test, although this is not a universal finding [40, 41]. The reasons for these apparent discrepancies are unknown but in our cohort there was significant variability in the 6MWD, which is likely to be influenced by a number of complex pulmonary and extra-pulmonary manifestations of disease. In addition, co-morbidities and psychological factors can have an effect on walking distance which were not investigated as part of this analysis. Desaturation during the 6MWT has also been linked with mortality in COPD patients [34, 42, 43]. On univariate analysis we found an association between desaturation and %LAA_<− 950_ and E/I MLD but not Pi10. On multivariate analysis this association only remained significant for %LAA_<− 950_. We also found that subjects who desaturated more than 5% on exertion had significantly lower FEV1% and higher %LAA_<− 950_ and on multivariate analysis, %LAA_<− 950_ was the only variable which independently predicted whether a subjects desaturated. We used > 5% to define significant desaturation as this has previously been linked to increasing mortality [43]. To the best of our knowledge the association between desaturation and CT parameters has not been previously investigated and our results suggest that emphysema, but not airways remodelling are directly linked to oxygen desaturation during exertion in COPD. This study does not provide any mechanistic explanations for this although a plausible suggestion would be the ventilation/perfusion mismatch that is caused by alveolar destruction seen in emphysema.

Few previous studies have investigated the link between CT parameters and biological markers of inflammation and microbiology. Although multiple studies have shown increased airway neutrophils in COPD, we found no association between any of the quantitative CT markers and sputum white cell differential. Previous studies have also demonstrated no link between emphysema and airway neutrophils [12, 32, 44, 45] and inconsistent results with sputum eosinophils [44, 46]. As opposed to this, a study using PET-CT demonstrated a relationship between pulmonary uptake of FDG, a marker of neutrophilic inflammation, and emphysema severity [47]. Prior work has also shown inconsistent results when investigating the link between CT parameters of gas trapping and airway neutrophil counts [12, 32], although these studies differed from ours as they utilised BAL sampling. Histological studies have also shown equivocal results regarding neutrophilia within the distal airways [48, 49]. The reason why our study and others fail to show a consistent link between neutrophils and emphysema or small airways disease is unknown, however it may be that the concentration of airway neutrophils at a single-time point is more influenced by other factors such as airway microbiology or smoking status. There was a suggestion of a negative relationship between emphysema and systemic inflammation, although the association was rather weak and so the relevance of this finding is unknown, which is further highlighted by a previous study showing increased fibrinogen but not CRP in emphysematous subjects [50].

We have previously shown in this cohort that colonisation of the airways with potentially pathogenic bacteria is important as it is related to exacerbations [17], making it vital to understand the underlying features which pre-dispose to this. We found few differences in CT parameters between subjects where bacteria were detected in sputum and those where it was not, by either culture or PCR. Few previous studies have investigated the link between structural changes identified on CT and microbiology, although one study supported our findings by demonstrating no difference in sputum bacterial culture between patients who had emphysema on CT and those who did not [46]. It is perhaps unsurprising that bacterial detection did not influence these structural changes as colonisation or invasion is likely to be due to abnormalities within the epithelial mucosal surface or immunological deficiencies that cannot be detected on CT. There are also obvious limitations on relying on sputum culture and PCR for bacterial detection and so further work is required to explore the relationship between the microbiome and structural changes within the lung.

There were a number of limitations to this study. Firstly, a number of different quantitative analysis techniques exist with which to measure each facet of structural lung disease, however we chose some of the most widely used and best-validated techniques. There is particular uncertainty for using Pi10 as a measure of airway wall dimensions, as simplifying the complexity of the bronchial tree into one number may be sub-optimal. Further work is required to understand which technique represents the best measure of airway wall dimensions in COPD. More recently, co-registration techniques such as parametric response mapping have been developed to estimate emphysema and small airways disease [51]. It has been proposed that this analysis method may be more accurate than those employed in this study although there is little direct evidence to support this currently. As is standard, we measured air trapping on CT by scanning subjects at maximal expiration. In some subjects, especially those with more severe COPD it may have been difficult to obtain residual volume which could have introduced errors. Furthermore, physiological and CT measurements of air trapping may not only reflect small airways disease but air trapping secondary to emphysema and therefore further work is required to validate CT measures by comparing them with more specific physiological measures of small airways disease. Another limitation is the multiple comparisons made in this study, however we found far more significant associations than would be expected by chance alone. For a CT study the sample size was relatively small, which may have limited the statistical power of the study, although the benefit of this was that it allowed us to perform substantial and in-depth phenotyping of the cohort. In addition, the results reported in this study were cross-sectional and some of the variables may vary over time and therefore further longitudinal studies are required to understand this.

## Conclusions

In conclusion, CT-derived measurements of emphysema and E/I MLD had independent associations with FEV1%, providing further evidence that both of these structural abnormalities are directly associated with the airflow obstruction that defines COPD. We also provided further validation for their use, as CT-measures of emphysema showed strong associations with gas transfer and CT-measure of gas trapping with RV. Furthermore, quantitative CT measures of emphysema have independent associations with desaturation on exertion, a measure which has been linked to mortality and therefore may help explain the mechanism by which emphysema is related to mortality. In contrast to this, quantitative CT parameters did not show any convincing associations with biological markers of disease. This suggests that while quantitative CT measures of emphysema and small airways disease are useful tools at reflecting pulmonary physiology and functional status they are less effective at reflecting disease biology and therefore may not be a useful tool in assessing disease activity.

## Additional file

Additional file 1: **Table S1**. Spearman’s correlation analysis between CT parameters and sputum markers (all sputum samples). **Table S2**. CT parameters in subjects according to sputum Bacterial PCR Detection. **Figure S1**. Scatterplots of (A) %LAA_<− 950_ against Pi10 (rho − 0.36***, *p* < 0.001) (B) E/I MLD against Pi10 (rho 0.18*, *p* 0.045) (C) %LAA_<− 950_ against E/I MLD (rho 0.47***, *p* < 0.001). (DOCX 74 kb)

## Abbreviations

%LAA_<− 950_: Lung voxels on the inspiratory scan with attenuation values below − 950 Hounsfield units
6MWD: Six-minute walk distance
6MWT: Six-minute walk test
AERIS: The Acute Exacerbation and Respiratory Infections in COPD study
COPD: Chronic obstructive pulmonary disease
CRP: C-reactive protein
CT: Computed tomography
E/I MLD: The ratio of the mean lung density, expiration/ inspiration
FEF75–25%: The forced expiratory flow at 25–75% of forced vital capacity
FEV1: Forced expiratory volume in 1 s
FVC: Forced vital capacity
MCAT: Moraxella catarrhalis
NTHI: Non-typeable Haemophilus influenza
PA: Pseudomonas aeruginosa
PCT: Pro-calcitonin
PPM: Potentially pathogenic bacteria
RV: Residual volume
SA: *Staphylococcus aureus*
SP: Streptococcus pneumonia
TLC: Total lung capacity
TLCO: Carbon monoxide transfer factor
