# Supplementary material for: Relationship of CT-quantified emphysema, small airways disease and bronchial wall dimensions with physiological, inflammatory and infective measures in COPD
Source: Respir Res. 2018 Feb 20;19:31. doi: 10.1186/s12931-018-0734-y (PMC5819274; doi:10.1186/s12931-018-0734-y)
Supplement: Supplementary file 1 — Table S1. Spearman’s correlation analysis between CT parameters and sputum markers (all sputum samples). Table S2. CT parameters in subjects according to sputum Bacterial PCR Detection. Figure S1. Scatterplots of (A) %LAA<− 950 against Pi10 (rho − 0.36***, p < 0.001) (B) E/I MLD against Pi10 (rho 0.18*, p 0.045) (C) %LAA<− 950 against E/I MLD (rho 0.47***, p < 0.001). (DOCX 74 kb) [file 12931_2018_734_MOESM1_ESM.docx]

Table S1 Spearman’s correlation analysis between CT parameters and sputum markers (all sputum samples)

|  | %LAA_<-950_ | E/I MLD | Pi10 |
| --- | --- | --- | --- |
| Sputum Differential |  |  |  |
| %neutrophils | 0.14 | 0.14 | -0.11 |
| %eosinophils | 0.11 | 0.02 | -0.06 |

N=75

Table S2 CT parameters in subjects according to sputum Bacterial PCR Detection

|  | Frequency | %LAA_<-950_ | E/I MLD | Pi10 |
| --- | --- | --- | --- | --- |
| Potentially Pathogenic Bacteria | | | | |
| PCR positive | 38 | 13.1 (20.0) | 0.91 (0.08) | 3.79 (0.11) |
| PCR negative | 60 | 11.2 (21.2) | 0.91 (0.06) | 3.81 (0.13) |
| P value | **-** | 0.796 | 0.531 | 0.266 |
| Haemophilus influenzae |  |  |  |  |
| PCR positive | 44 | 13.9 (25.4) | 0.92 (0.08) | 3.79 (0.09) |
| PCR negative | 54 | 11.2 (19.7) | 0.91 (0.06) | 3.80 (0.12) |
| P value | - | 0.963 | 0.748 | 0.632 |
| Moraxella catarrhalis |  |  |  |  |
| PCR positive | 14 | 10.9 (26.8) | 0.89 (0.11) | 3.78 (0.12) |
| PCR negative | 84 | 13.1 (19.5) | 0.91 (0.06) | 3.80 (0.12) |
| P value | - | 0.605 | 0.446 | 0.281 |
| Streptococcus pneumoniae | | | | |
| PCR positive | 13 | 12.8 (10.8) | 0.89 (0.08) | 3.78 (0.12) |
| PCR negative | 85 | 11.5 (24.9) | 0.92 (0.07) | 3.80 (0.12) |
| P value | - | 0.718 | 0.141 | 0.238 |
| Staphylococcus aureus |  |  |  |  |
| PCR positive | 7 | 16.7 (19.8) | 0.92 (0.11) | 3.76 (0.07) |
| PCR negative | 91 | 12.1 (20.7) | 0.91 (0.07) | 3.80 (0.12) |
| P value | - | 0.923 | 0.799 | 0.081 |
| Pseudomonas aeruginosa |  |  |  |  |
| PCR positive | 6 | 21.4 (27.5) | 0.95 (0.06) | 3.83 (0.16) |
| PCR negative | 92 | 11.8 (20.1) | 0.91 (0.07) | 3.83 (0.16) |
| P value | - | 0.358 | **0.032** | 0.563 |

Frequency given as number of subjects. For CT parameters, values represent medians and IQR. *P<0.05 using Mann Whitney U test.

Figure S1 Scatterplots of (A) %LAA_<-950_ against Pi10 (rho -0.36***, p<0.001) (B) E/I MLD against Pi10 (rho 0.18*, p 0.045) (C) %LAA_<-950_ against E/I MLD (rho 0.47***, p<0.001)
